# Mobility and cognition at admission to the nursing home – a cross-sectional study

**DOI:** 10.1186/s12877-018-0724-4

**Published:** 2018-01-30

**Authors:** Karen Sverdrup, Sverre Bergh, Geir Selbæk, Irene Røen, Øyvind Kirkevold, Gro Gujord Tangen

**Affiliations:** 1 Norwegian National Advisory Unit on Ageing and Health, Vestfold Hospital Trust, Tønsberg, Norway; 2 Department of Geriatric Medicine, Oslo University Hospital, Oslo, Norway; 3 Centre for Old Age Psychiatric Research, Innlandet Hospital Trust, Ottestad, Norway; 4 Faculty of Medicine, University of Oslo, Oslo, Norway; 5 Department of Health Science, Norwegian University of Science and Technology, Gjøvik, Trondheim, Norway

**Keywords:** Mobility, Life space, Cognition, Dementia, Long-term care, Nursing home residents

## Abstract

**Background:**

Earlier studies show that the main reasons for admission to long-term nursing home care are cognitive impairment and functional impairments of activities of daily life. However, descriptive evidence of mobility is scant. The aims of this study were to describe mobility at admission to nursing homes and to assess the association between mobility and degree of dementia.

**Methods:**

We included 696 residents at admission to 47 nursing homes in Norway. Inclusion criteria were expected stay for more than 4 weeks and 65 years or older. In addition, younger residents with dementia were included. Residents with life expectancy shorter than six weeks were excluded. Mobility was assessed using the Short Physical Performance Battery (SPPB) and the Nursing Home Life Space Diameter (NHLSD). The Clinical Dementia Rating Scale (CDR) was used to describe the degree of dementia. The associations between mobility and degree of dementia was analysed using the Chi-square and the Kruskal-Wallis test (KW-test). When the KW-test indicated a statistical significant difference, we proceeded with planned group comparisons with the Mann-Whitney U-test. In addition, we performed multiple linear regression analyses to control for potential confounders.

**Results:**

Forty-three percent of the residents were not able to perform the balance test in SPPB. Twenty-four percent of the residents were not able to walk four meters, while only 17.6% had a walking speed of 0.83 m/s or higher. Sixty-two percent of the residents were not able to rise from a chair or spent more than 60 s doing it. The median score on NHLSD area was 22 (IQR 17) and the median score on NHLSD dependency was 36 (IQR 26). Residents with severe dementia had significantly lower levels of mobility than residents with moderate dementia. Cognitive function was associated with SPPB and NHLSD dependency in the adjusted models.

**Conclusion:**

Nursing home residents form a frail, but heterogeneous group both in terms of cognition and mobility at admission. Mobility was negatively associated with cognitive function, and residents with severe dementia had significantly lower levels of mobility than residents with moderate dementia.

## Background

The rapidly aging population has made the role of long-term care increasingly important worldwide. The present long-term care systems are under strain and the total incidence of people in need of long-term care is expected to rise in the coming years [1]. Nursing homes (NH) are the main providers of institutional long-term care in Norway [2]. The main reasons for admission to NH are cognitive impairment and dementia, and functional impairments of activities of daily life [1, 3, 4]. In Norway, the prevalence of dementia in NH residents has increased the past decades and as many as 84.3% of NH residents suffer from dementia [5]. However, dementia does not only lead to a decline in cognitive functions but also to impairment in mobility [6]. Mobility, the ability to move independently and safely from one place to another [7], is essential for all parts of activities of daily life [8]. To be able to maintain and improve mobility in NH residents it is important to identify underlying aspects of mobility, such as balance, gait and lower limb strength. In addition, there may be a gap between physical capacity and physical activity in NH residents, and it is important to gain information about the residents’ activity range and frequency, known as life space [9].

Several of the published studies providing descriptive information on NH residents’ mobility are results from baseline assessments of randomized controlled trials (RCT) [10–12]. However, one should be cautious to draw conclusions regarding mobility from such studies, as there is a substantial risk of selection bias where residents with a positive attitude towards exercise are more likely to participate. Strict inclusion criteria may also limit the generalizability of the descriptive results from RCT studies. There are also observational studies describing mobility in the NH population, but either the sample size is small [13, 14] or the study took place several years ago [15]. To our knowledge, only a few studies have described life space in NH residents, and interpretation of them is difficult as they used different outcome measures [9, 16]. Further, the studies that provide descriptive information on mobility in NH residents are carried out after various length of stay in the NH or don’t specify at what time-point during the residents’ stay at the NH the assessments were carried out [9, 13–16]. Throughout, these studies report that nursing home residents are frail but heterogenous. Mobility is associated with health status and quality of life [8] and to increase knowledge of mobility at time of admission to NH care is imperative.

In home-dwelling older people, high levels of mobility are associated with better cognitive functioning and a linear relationship between mobility and cognition is observed [6, 17–19]. NH residents are considered as the physically frailest in our population, and in addition to dementia other conditions such as arthritis, osteoporosis, stroke, diabetes and cardiovascular diagnoses are common and likely to entail mobility limitations [20]. The coexistence of several diagnoses among NH residents makes it hard to transfer the existing knowledge about mobility and cognition in home-dwelling elderly to NH residents. To date, there are few reports on the relationship between mobility and degree of dementia in NH residents. However, there are studies reporting that the level of mobility in NH residents decreases in line with deterioration in cognition [10, 21, 22].

Taken together, there is currently a lack of knowledge about mobility and associations with degree of dementia in NH residents at time of admission. The aims of this study were to describe mobility and life space at admission to NH care, and to explore the associations between degree of dementia and mobility.

## Methods

### Study design and setting

This study is a multicentre, cross-sectional study from the baseline assessments of the Recourse Use and Disease Course in dementia – Nursing Home (REDIC-NH) study. In all, 47 NH from 35 municipalities within four counties in south of Norway participated in the study. Inclusion took place from March 2012 through November 2014. The REDIC-NH study methods have been described in detail in an earlier publication [23].

### Participants

Participants were consecutively included at admission to the NH. Residents were eligible for inclusion if they had an expected stay at the NH of more than four weeks and were 65 years or older. Younger persons with established dementia were also included. Residents with a life expectancy of less than six weeks as judged by the nursing home physician, the responsible nurse and the primary caregiver were excluded. Written and oral information about the study was given by a project team member, the department manager at the NH or the nursing home physician. Participation was based on consent given by the resident, or by the resident’s next of kin when the resident him/herself was not able to consent to participation. Ability to consent in the study was decided by the NH staff, including the physician. The Regional Ethics committee for Medical Research in South-Eastern Norway approved the study (2011/1738a).

### Procedure

The data on mobility and cognition was collected by health workers in the NH, in collaboration with research nurses. The research nurses completed a five-day training program and the health workers in the NH participated in a two-day training program prior to the first assessments. In addition to the training program the assessors were provided with a written guideline for completion of the case report [23].

### Measurements

The Short Physical Performance Battery (SPPB*)* was used to assess mobility. The SPPB is a clinical performance-based screening test of physical function and mobility in older adults [24]. The SPPB includes three tests that assess static balance, gait speed and lower limb strength. Each test is scored from 0 to 4, and the total score is 0–12 points. Higher score indicates higher functional status, thus better mobility [25].

The Nursing Home Life Space Diameter (NHLSD) was used to measure life space. The NHLSD assesses the extent and frequency of mobility, and is completed by a health care worker [9]. Four different ranges (extent) are described and points from 1 to 4 are given. The innermost range (within his or her room) is 1 point and the outermost (outside the facility) is given 4 points. Frequency for each range is scored from 0 to 5, where 0 is never and 5 is more than three times a day. Independency or need for assistance during mobility is also noted. If independency of movement the score is multiplied by two. Scoring range is 0–100; higher score indicates larger range of mobility and greater independence [9].

The Clinical Dementia Rating Scale (CDR) was used to describe the degree of cognitive impairment and dementia [26]. The CDR is a global rating tool where performance on six domains of cognitive and functional performance are rated. Based on an algorithm which gives precedence to the domain memory, a CDR score of 0, 0.5, 1, 2 or 3, indicating no, questionable, mild, moderate and severe dementia, respectively, is produced. In line with previous studies, a CDR score of 1 or more indicated dementia [5]. CDR can also be scored as sum of boxes (CDR-SOB), ranging from 0 to 18, where higher score indicates more impaired cognitive function [27].

### Demographic factors

Sociodemographic characteristics and clinical information include gender, age, years of education, marital status, body mass index (BMI), comorbidity and general physical health. Comorbidity was assessed with the Charlson comorbidity index (CCI) [28]. CCI includes 18 different conditions. Based on information from the patients’ journals each condition is scored yes/no which gives a count of total number of comorbid conditions. The General Medical Health Rating (GMHR) scale was used to assess general physical health. The GMHR is a one-item, global rating scale with four categories (excellent, good, fair, poor) [29].

### Statistical analysis

Data are presented with numbers and percentages for categorical variables, with means and standard deviation (SD) for normally distributed continuous data, and with median and interquartile range (IQR) for continuous data with skewed distribution. Several of the variables have missing data, and the number of cases will vary between the different analyses. We used the Chi-square test to compare categorical data, and the Kruskal-Wallis test (KW-test) to compare continuous data with skewed distribution. For the group comparisons, we dichotomized the chair stand variable into > 60 s or unable to rise from chair (category 0) and less than 60 s (category 1–4). When the KW-test indicated a significant difference, we proceeded with planned group comparisons with the Mann-Whitney U-test. Based on the linear relationship between mobility and cognition observed in home-dwelling elderly [6, 17–19], we performed pre-planned comparisons between groups of increasing degree of dementia. For these analyses, we applied the Bonferroni adjustment to the alpha value of 0.05, and the significance level was therefore set at 0.017. For all other statistical tests, the *p*-value was set at 0.05. We performed multiple regression analyses of the relationships between cognition (using CDR-SOB) and the three main outcome variables SPPB, NHLSD area and NHLSD dependency. We adjusted for age, gender, marital status and GMHR. For these analyses, we dichotomized marital status (unmarried/divorced/widowed and married/partner) and GMHR (excellent/good and fair/poor). CDR-SOB was entered into the models in a separate block, to provide information of its individual contribution to the explained variance of the models. We checked correlations between the independent variables for collinearity, and also looked at the residual plots to ensure that the model assumptions were not violated. All these statistical analyses were conducted in IBM SPSS Statistics version 23. In addition, we performed multilevel models with nursing home modelled as a random effect using xtmixed in Stata. The models with nursing home as random effect did not provide a better fit to the data and remained largely unchanged from the models not including this random effect. We therefore only present the multiple regression models.

## Results

Table 1 shows the characteristics of the study population. A total of 696 residents were included, and 63.9% were women. The mean age was 84.4 years (SD 7.5) and 56.2% of the residents were widowed. Based on the CDR score 86.4% had dementia (CDR score 1–3) at admission to the NH. Fifteen residents (2.2%) had no dementia (CDR score 0) and 78 (11.4%) had questionable dementia (CDR score 0.5). These two groups are therefore treated as one group (no OR questionable dementia) in the analyses.

**Table 1:** Baseline characteristics, mobility performance and degree of dementia for the nursing home residents

	Total sample (*N* = 696)	
Participants per NH [median (IQR)]	10	(14)
Female gender [n (%)]	445	(63.9)
Age [mean (SD)]	84.4	(7.5)
BMI [mean (SD)]	23.9	(4.5)
Education (y) [median (IQR)]	8	(2)
Marital status [n (%)]	*n* = 687	
Unmarried	60	(8.7)
Married/ Partner	209	(30.4)
Widowed	386	(56.2)
Divorced	32	(4.7)
CCI, sum [median (IQR)]	2	(2)
GMHR [n (%)]	*n* = 666	
Excellent	32	(4.8)
Good	285	(42.8)
Fair	270	(40.5)
Poor	79	(11.9)
SPPB SUM [median (IQR)]	4	(6)
SPPB Balance [n (%)]	*n* = 589	
0. < 10 s or can’t perform	251	(42.6)
1. Side by side	86	(14.6)
2. Semi tandem	101	(17.1)
3. Tandem 3–9.99 s	54	(9.2)
4. Tandem	97	(16.5)
SPPB Gait [n (%)]	*n* = 636	
0. Can’t perform	150	(23.6)
1. < 0.46 m/s	157	(24.7)
2. 0.46–0.64 m/s	112	(17.6)
3. 0.65–0.83 m/s	105	(16.5)
4. > 0.83 m/s	112	(17.6)
SPPB Chair stand [n (%)]	*n* = 570	
0. > 60 s or can’t perform	356	(62.5)
1. > 16.7 s	82	(14.4)
2. 13.70–16.99 s	42	(7.4)
3. 11.20–13.69 s	34	(6.0)
4. < 11.19 s	56	(9.8)
NHLSD area [median (IQR)]	22	(17)
NHLSD dependency [median (IQR)]	36	(26)
CDR [n (%)]	*n* = 685	
0. No dementia	15	(2.2)
0.5. Questionable dementia	78	(11.4)
1. Mild dementia	178	(26.0)
2. Moderate dementia	276	(40.3)
3. Severe dementia	138	(20.1)
CDR-SOB [mean (SD)]	10.3	(4.3)

*NH* Nursing Home, *IQR* Interquartile Range, *SD* Standard deviation, *BMI* Body Mass Index, *CCI* Charlson Comorbidity Index, *GMHR* General Medical Health Rating, *SPPB* Short Physical Performance Battery, *NHLSD* Nursing Home Life Space Diameter, *CDR* Clinical Dementia Rating. *CDR-SOB* Clinical Dementia Rating sum of boxes

% = valid percent without missing

SPPB SUM: range 0–12, higher score indicates minimal mobility limitations/higher physical functioning

NHLSD area: range 0–50, higher score indicates wider life space

NHLSD dependency: range 0–100, higher score indicates independency and wider life space

CDR-SOB: range 0–18, higher score indicates more impaired cognitive function

The median total score on SPPB was 4 points (IQR 6). Close to half of the residents were not able to perform the balance test in SPPB (42.6%), and only 16.5% of the residents could achieve the highest score (Tandem). On the gait test in SPPB 23.6% of the residents were not able perform the task, while only 17.6% had a walking speed of 0.83 m/s or higher. Roughly two thirds of the residents used more than one minute or were not able to perform the chair stand test in SPPB (62.5%), and 9.8% of the residents could achieve the highest score (< 11.19 s). The median score on NHLSD area was 22 (IQR 17) and the median score on NHLSD dependency was 36 (IQR 26). (Table 1).

There was a significant difference in score on all the mobility outcome measures per the degree of dementia (Table 2). Planned group comparisons showed that the group of residents with severe dementia had significantly lower mobility scores measured by both SPPB as well as life space than residents with moderate dementia (Table 3).

**Table 2:** Associations between degree of dementia and mobility

	No/ questionable dementia (*N =* 93)		Mild dementia (*N* = 178)		Moderate dementia (*N* = 276)		Severe dementia (*N* = 138)		*P-*value
SPPB SUM [median (IQR)]	4	(5)	4	(6)	4	(6)	2	(4)	< 0.001^a^
SPPB Balance [n (%)]									0.002^b^
0. < 10 s or Can’t perform	35	(49.3)	57	(35.6)	89	(36.8)	66	(59.5)	
1. Side by side	13	(18.3)	25	(15.6)	36	(14.9)	12	(10.8)	
2. Semi tandem	8	(11.3)	27	(16.9)	53	(21.9)	12	(10.8)	
3. Tandem 3–9.99 s	5	(7.0)	13	(8.1)	26	(10.7)	10	(9.0)	
4. Tandem	10	(14.1)	38	(23.8)	38	(15.7)	11	(9.9)	
SPPB Gait [n (%)]									< 0.001^b^
0. Can’t perform	27	(32.5)	43	(25.6)	36	(14)	43	(35.8)	
1. < 0.46 m/s	20	(24.1)	36	(23.4)	68	(26.5)	28	(23.3)	
2. 0.46–0.64 m/s	12	(14.5)	29	(17.3)	49	(19.1)	21	(17.5)	
3. 0.65–0.83 m/s	15	(18.1)	30	(17.9)	53	(20.6)	7	(5.8)	
4. > 0.83 m/s	9	(10.8)	30	(17.9)	51	(19.8)	21	(17.5)	
SPPB Chair stand [n (%)]									< 0.001^b^
0. > 60 s or can’t perform	45	(69.2)	93	(62.0)	125	(53.4)	86	(76.1)	
1. Can perform in < 60 s	20	(30.8)	57	(38.0)	109	(46.6)	27	(23.9)	
NHLSD area [median (IQR)]	18	(15)	22	(16)	22	(19)	15	(19)	0.006^a^
NHLSD dependency [median (IQR)]	30	(26)	37	(22)	37	(26)	30	(27)	< 0.001^a^

*SPPB* Short Physical Performance Battery, *IQR* Interquartile Range, *NHLSD* Nursing Home Life Space Diameter

SPPB SUM: range 0–12, higher score indicates minimal mobility limitations/higher physical functioning

NHLSD area: range 0–50, higher score indicates wider life space

NHLSD dependency: range 0–100, higher score indicates independency and wider life space

% = valid percent without missing

^a^Kruskal-Wallis test, ^b^Chi-square test

**Table 3:** Planned group comparisons of mobility between degree of dementia

	(1) No/ questionable dementia vs. Mild dementia	(2) Mild dementia vs. Moderate dementia	(3) Moderate dementia vs. Severe dementia
SPPB SUM	*p* = 0.27^a^	*p* = 0.15^a^	*p* < 0.001^a^
SPPB Balance	*p* = 0.22^b^	*p* = 0.26^b^	*p* = 0.002^b^
SPPB Gait	*p* = 0.54^b^	*p* = 0.06^b^	*p* < 0.001^b^
SPPB Chair stand	*p* = 0.35^b^	*p* = 0.11^b^	*p* < 0.001^b^
NHLSD area	*p* = 0.22^a^	*p* = 0.10^a^	*p* = 0.003^a^
NHLSD dependency	*p* = 0.013^a^	*p* = 0.36^a^	*p* = 0.001^a^

*SPPB* Short Physical Performance Battery, *NHLSD* Nursing Home Life Space Diameter

^a^Mann-Whitney U test, ^b^Chi-square test

In the multiple regression analyses, degree of cognitive impairment (CDR-SOB) was significantly associated with SPPB (*p* = 0.015) and with NHLSD dependency (*p* = 0.01), but not with NHLSD area (*p* = 0.07) (Table 4). Adjusted explained variance of these three models was between 12% and 20%, and CDR-SOB contributed with 1% of the variance in the models with SPPB and NHLSD dependency. There were no significant differences in age, gender, marital status and GMHR between participants included in the regression analyses and participants excluded because of missing data.

**Table 4:** Multiple linear regression analyses of the relationship between cognition and mobility

SPPB sum score (*n* = 486)						
	Crude estimates			Adjusted estimates (Adj. R^2^ = 0.197)		
	B	95% CI	*p*	B	95% CI	*p*
Age	−0.10	−0.14, − 0.06	< 0.001	− 0.11	−0.14, − 0.07	< 0.001
Gender	− 0.04	−0.69, 0.61	0.90	0.14	−0.49, 0,77	0.65
Marital status^a^	−0.18	−0.87, 0.51	0.61	−0.62	−1.30, 0.05	0.07
GMHR^b^	2,80	2.22, 3.38	< 0,001	2.80	2.23, 3.38	< 0.001
CDR-SOB	−0.08	−0.16, − 0.004	0.04	− 0.09	−0.16, − 0.12	0.015
NHLSD (*n* = 576) - area						
	Crude estimates			Adjusted estimates (Adj. R^2^ = 0.12)		
	B	95% CI	*p*	B	95% CI	*p*
Age	−0.47	−0.60, − 0.34	< 0.001	− 0.48	−0.62, − 0.34	< 0.001
Gender	0.40	−1.71, 2.51	0.71	0.25	−1.88, 2.38	0.82
Marital status^a^	0.39	−1.82, 2.59	0.73	−1.48	−3.78, 0.83	0.21
GMHR^b^	6.07	4.05, 8.09	< 0.001	6.04	4.07, 8.01	< 0.001
CDR-SOB	−0.09	−0.33, 0.15	0.45	−0.21	−0.44, 0.02	0.07
NHLSD (*n* = 576) - dependency						
	Crude estimates			Adjusted estimates (Adj. R^2^ = 0.14)		
	B	95% CI	*p*	B	95% CI	*p*
Age	−0.75	−0.98, − 0.52	< 0.001	− 0.79	−1.03, − 0.54	< 0.001
Gender	2.63	−1.10, 6.36	0.17	3.33	−0.44, 7.10	0.08
Marital status^a^	−0.26	−4.19, 3.66	0.90	−4.02	−8.10, 0.06	0.054
GMHR^b^	12.36	8.79, 15.92	< 0.001	12.7	9.22, 16.20	< 0.001
CDR-SOB	−0.34	−0.76, 0.08	0.11	−0.53	−0.94, − 0.12	0.01

*SPPB* Short Physical performance Battery, *CI* Confidence Interval, *GMHR* General Medical Health Rating, *NHLSD* Nursing Home Life Space Diameter, *CDR-SOB* Clinical Dementia Rating sum of boxes

^a^Marital status dichotomized into unmarried/divorced/widowed (0) vs. married/partner (1)

^b^GMHR dichotomized into fair/poor (0) vs. excellent/good (1)

## Discussion

At admission to the NH, residents in this study formed a heterogeneous group. Lower levels of mobility were associated with more severe cognitive impairment. Overall, residents with severe dementia had significantly lower levels of mobility than residents with moderate dementia.

All our participants were included at the time of admission to a NH. However, most other studies that provide descriptive information in NH residents are carried out at various time-points during stay at the NH or don’t specify at what time of the residents’ stay the measurements were carried out. In lack of studies carried out at admission, we have chosen to compare our results to the NH population in general.

The overall score on the SPPB indicates that the residents had moderate to severe physical limitations and low levels of mobility [25]. Close to half of the residents were not able to complete the balance test, which indicates slightly lower levels of balance abilities than previously described [13, 15]. As many as 62.5% of the residents were not able to rise from a chair five times, or used more than one minute to complete the test, which is in line with the study from Grönstedt et al. [10]. A recent systematic review reports the average walking speed in nursing home residents as 0.47 m/s [30]. In the present study 23.6% of the residents were not able to complete the four-meter walk test and 58.8% of residents had a walking speed between 0.46 and 0.83 m/s. The residents’ life space was within their room or their own unit and beyond their unit once a week (NHLSD area 22). Regarding their *independent* mobility, residents moved within their room and unit daily, and outside their unit less than weekly (NHLSD dependency 36). These results are consistent with the results in previous studies [10, 16].

In bivariate analyses, we found that residents with severe dementia had significantly poorer performances on mobility and more limited life space than residents with moderate dementia. In the adjusted models, degree of cognitive impairment was significantly associated with mobility, which is consistent with previous studies [10, 21, 22]. However, it is important to acknowledge that cognition only contributed with 1% of the explained variance of the mobility outcomes. In studies of home-dwelling persons with dementia, cognitive function has explained up to 13% of the variance in mobility outcomes [6]. So, the relationship between mobility and cognition appears to be more complex in nursing home residents than in home-dwelling persons with dementia.

Furthermore, residents with no or questionable dementia were more dependent on assistance from others during mobility than residents with mild dementia. The main reason for admission to a nursing home is cognitive impairment and impairment in ability to carry out activities of daily life [1, 3, 4]. In addition to cognitive impairment, conditions such as arthritis, osteoporosis, stroke, diabetes and cardiovascular diagnoses are common in NH residents and likely to entail mobility limitations [20]. Thus, residents who don’t suffer from dementia are most likely admitted due to various diseases that entail mobility limitations. In contrast to findings from studies of home-dwelling older people we did not find a gradual deterioration of mobility with increasing degree of dementia [6, 18]. Since residents are admitted due to a combination of reasons, the linear deterioration of mobility in line with increasing degree of dementia might not be as evident as in home-dwelling elderly. Still it is important to recognise the vulnerability of the residents with severe dementia as they displayed very low levels of mobility at admission. When planning future care systems, it is evident that these residents require additional resources compared to residents with less severe dementia.

Even though the residents had moderate to severe physical limitations and low levels of mobility at admission, they formed a heterogeneous group regarding overall mobility and life space, expressed by the large interquartile ranges. This result is in line with previous studies of residents with various duration of NH stay [10, 15, 21, 22, 31]. The heterogeneity indicates the importance of registering level of mobility already at admission to a NH to be able to provide individually tailored services and care. Registering the level of mobility at admission to NH is important to be able to implement interventions to improve or prevent further decline in mobility. Several studies have examined the effects of various exercise and physical activity interventions on mobility and cognition in NH residents, with promising results [32, 33]. However, the overall low levels of mobility and high percentage of residents with moderate and severe degree of dementia demonstrated in our study indicate that further studies on effect of intervention including residents on the entire continuum of mobility and cognition are needed. The level of mobility and degree of dementia described in our study is important to recognise when considering staffing in NH. Our study shows that residents’ life space primarily was within their room or unit. This knowledge should be taken into consideration when planning the structure of NH, signifying that residents need assistance for reaching areas, services and activities provided outside their units or even outside their room. In addition, these results have implications for where organized activities should take place and that interventions should aim at facilitating the ability of residents to increase their life space to be able to attend activities outside their room and unit.

Some limitations should be considered. In particular, the group of no or questionable dementia was relatively small, and the study is likely to be underpowered to detect small differences in mobility between no or questionable dementia and mild dementia. The registration rate on almost all variables was above 80% which we consider as acceptable, however, the rate was lower on the SPPB. Just 55.9% in the small group of the residents with no or questionable dementia had complete score on SPPB. One possible explanation is that the residents may have had very poor mobility so that the health workers did not consider the SPPB as a feasible test (although “can’t perform” is an option in the test manual). We do therefore not consider the missing data as random, and we have chosen not to use imputation techniques. Consequently, we must interpret the results related to the group of no or questionable dementia with caution. Another limitation is that gait speed was registered as a categorical variable in the SPPB. The lack of gait speed as a continuous variable limits the use of established cut offs and limits the accuracy to which we can compare our results to other studies.

We consider the multicentre approach as a strength of this study. However, local practices and multiple assessors with various professional background and previous experience with the assessment tools, can affect how the assessments are carried out. To standardize the assessments across the different inclusion sites, comprehensive training for all the assessors involved was provided and the case report form contained a guideline on how to complete the assessments. We believe that this has contributed to standardization of assessments throughout the study. Further, the large sample size, broad inclusion criteria and use of performance-based outcomes for mobility should be considered as strengths of this study. To the best of our knowledge this is the first study to describe mobility using performance-based tests at admission to a NH. The cross-sectional design precludes any interpretation regarding the direction of the associations. Further epidemiological studies with a prospective approach are needed to explore the trajectory of mobility in NH residents.

## Conclusion

NH residents form a frail, but heterogeneous group both in terms of cognition and mobility. We observed a relationship between mobility and cognition, however not as clear as in previous studies of home-dwelling persons. Mobility was negatively associated with cognitive function, and residents with severe dementia had significantly lower levels of mobility than residents with moderate dementia. To be able to provide individually tailored care, and implement interventions that promote participation in activities of daily life and quality of life in NH, our results highlight the importance of mapping out the residents’ mobility at admission to a NH.

## Abbreviations

BMI: Body mass index
CCI: Charlson comorbidity index
CDR: Clinical dementia rating scale
CDR-SOB: Clinical dementia rating scale sum of boxes
GMHR: General Medical Health Rating
NH: nursing home
NHLSD: Nursing home life space diameter
REDIC-NH: Recourse use and disease course in dementia – nursing home
SPPB: Short physical performance battery
